# Adherence to Levothyroxine Treatment Among Patients With Hypothyroidism: A Northeastern Italian Survey

**DOI:** 10.3389/fendo.2018.00699

**Published:** 2018-11-23

**Authors:** Carlo Cappelli, Roberto Castello, Fiorella Marini, Agostino Paoletta, Massimo Marchetti, Maura Saullo, Alessandra Cristiano, Ilenia Pirola, Elena Gandossi, Alberto Ferlin, Maurizio Castellano

**Affiliations:** ^1^Department of Clinical and Experimental Sciences, SSD Medicina ad Indirizzo Endocrino-Metabolico, University of Brescia, ASST Spedali Civili di Brescia, Brescia, Italy; ^2^General Medicine and Endocrinology, University Hospital, Azienda Ospedaliera Universitaria Integrata Verona, Verona, Italy; ^3^Endocrinology Outpatient Service, Azienda ULSS 6 Euganea, Cittadella, Italy; ^4^General Medicine and Endocrinology, Ospedale Bassano del Grappa, Bassano del Grappa, Italy

**Keywords:** hypothyroidism, levothyroxine formulation, liquid levothyroxine, thyrotropin, adherence treatment, replacement therapy

## Abstract

**Background:** A significant number of patients show sub-optimal adherence to levothyroxine (LT4) therapy, mainly because they have to postpone their breakfast by at least 30 min. The aim of this observational cross-sectional study was to assess the therapeutic compliance of patients on LT4 treatment and to verify the preference of a lifetime treatment in tablet or liquid form.

**Patients and Methods:** Ambulatory care patients aged 18 years or older, affected by hypothyroidism and on LT4 treatment (in tablet or liquid form) were administered the eight-item Morisky Medication Adherence Scale (MMAS-8). The MMAS-8 questionnaire was supplemented with 3 further items to specifically evaluate preference between tablet and liquid forms of LT4 for lifetime treatment.

**Results:** A total of 320 patients (272 female), median age 47.9 ± 15.6 years (range, 20–78 years), completed the MMAS-8 questionnaire. Eighty-seven percent of the participants were adhering to their treatment for both tablet and liquid LT4 formulations, although significant differences emerged. Patients on LT4 tablets forgot to take their medication more frequently (*p* < 0.001), felt hassled about sticking to their treatment plan (*p* < 0.001), and had difficulty remembering to take all their medication(s) (*p* < 0.001) than those on liquid LT4 treatment.

**Conclusions:** Adherence to LT4 treatment was high for both tablet and liquid formulations. Taking LT4 at breakfast was the most convenient option for most patients.

## Introduction

Hypothyroidism is a common clinical problem worldwide requiring life-long thyroid hormone replacement therapy for most patients. Studies in Northern Europe, Japan, and the USA have found that its prevalence ranges between 0.6 and 12 per 1,000 women and between 1.3 and 4.0 per 1,000 men (1–4). In addition, the prevalence of hypothyroidism, mainly due to autoimmune thyroiditis, increases with increasing age (5).

Levothyroxine (LT4) is a reliable and commonly prescribed drug to treat thyroid disease. The aim of substitution therapy is to resolve symptoms and signs of hypothyroidism and maintain the serum thyrotropin (TSH) concentration within a narrow range, which indicates successful treatment. Even though the management of hypothyroidism is generally considered straightforward, several cross-sectional surveys of patients receiving LT4 have shown that a large percentage of patients (40–48%) are over- or undertreated (2, 6). Moreover, a significant number of patients showed sub-optimal adherence to LT4 therapy, mainly because they had to postpone their breakfast by at least 30 min (7).

During the past few years, various LT4 formulations (tablets, soft-gel capsules, and liquid solutions) have become available for clinical use (8). These formulations have been shown to be more efficacious in many cases of refractory hypothyroidism, such as among patients with many gastrointestinal diseases, including *Helicobacter pylori* infection, celiac disease, atrophic body gastritis, but also due to some medications (9). In addition, in patients with subclinical hypothyroidism and those with central hypothyroidism, LT4 liquid formulations seem to be more effective in restoring euthyroidism than tablets (10, 11). Moreover, recent clinical trials have clearly shown, in particular for liquid forms, that these formulations are able to circumvent the optimal absorption of LT4 arising with concomitant ingestion with food, particularly at breakfast (12, 13). This finding may represent a significant advantage for the liquid LT4 formulation to maximize therapeutic compliance.

To date, adherence to LT4 therapy among patients with hypothyroidism is still not well characterized. The present prospective observational study aims to assess the therapeutic compliance of patients on LT4 treatment and to verify the preference for lifetime treatment in tablet or liquid form.

## Materials and methods

In this observational cross-sectional study, 320 patients were recruited from various public hospitals in Brescia, Verona, Padova, and Bassano del Grappa, representing a large area in northeast Italy. All patients had to be 18 years or older, affected by hypothyroidism and on LT4 substitution treatment either as a tablet or liquid in a single-dose vial.

All participants were administered the eight-item Morisky Medication Adherence Scale (MMAS-8), a structured self-reporting medication adherence measure, to identify the behavior of patients with regard to adherence to prescribed medications. MMAS-8 consists of 7 yes/no items and one 5-point Likert scale measuring specific medication-taking behavior such as “forgetfulness,” “feeling hassled about sticking to the treatment plan,” or “stopping the regimen because the medication makes the patient feel worse” (14). The MMAS-8 questionnaire was supplemented with 3 further items to specifically evaluate medication preferences between tablet and liquid LT4 formulations for lifetime treatment.

The MMAS-8 score (range, 0–8) was calculated and trichotomized into low (score <6), medium (score 6 or 7), and high (score ≥8) adherence (14).

The study was approved by an independent Institutional Review Board and conducted in compliance with the Declaration of Helsinki and the Good Clinical Practice Guidelines of the International Conference on Harmonisation. All participants provided written informed consent.

### Statistical analysis

All data were collected in an electronic case report database. Comparisons between groups and difference between proportions were calculated using the χ^2^ test for categorical variables and ANOVA for quantitative variables, as appropriate. A two-tailed *p*-value < 0.05 was considered statistically significant. Statistical analyses were performed using SPSS 20.0 software (SPSS, Inc., Evanston, IL, USA).

## Results

A total of 320 patients (272 female), mean age of 47.9 ± 15.6 years (range, 20–78 years), completed the MMAS-8 questionnaire. One hundred and sixty-one patients (87% female) were on LT4 replacement therapy in tablet form. Among these patients, 11 (6.8%) were taking LT4 for <1 year, 31 (19.3%) for 1–5 years, 56 (34.8%) for 5–10 years, and 63 (39.1%) for more than 10 years. One hundred and fifty-nine patient (83% female) were on a liquid LT4 formulation, 19 (11.9%) for <1 year, 65 (40.9%) for 1–5 years, and 75 (47.2%) for 5–10 years. Gender was equally distributed among patients on tablet and liquid LT4 formulations (140/21 and 132/27 female/male, respectively, *p* = 0.203). Participants on tablet LT4 replacement therapy were older than those on liquid LT4 formulations (56.2 ± 15.3 years [range, 20–78 years] vs. 39.5 ± 10.8 years [range, 22–76 years], respectively, *p* < 0.0001). The responses to the MMAS-8 questionnaire are reported in Table 1. Using these cut-off points mentioned earlier, this study population had 1.9% low adherers (1.2% on tablets and 2.5% on liquid), 10.9% medium adherers (10.6 and 11.3%, respectively), and 87.2% high adherers (88.2 and 86.2%, respectively). The patients on tablet LT4 forgot to take their medication more frequently than those on liquid LT4 treatment (64.5% vs. 34.5%, *p* < 0.001), felt hassled about sticking to their treatment plan (72% vs. 39.6%, *p* < 0.001) and had difficulty (usually or always) remembering to take all their medication(s) (67% vs. 26.2, *p* < 0.001), respectively. A subset analysis was performed matching patients one to one in accordance to age. One hundred and ninety-seven (86% female) patients, median age 50.3 ± 17.0 years (range, 20–78 years) were evaluated. The results were similar to those obtained for all participants; specifically, the 79.3 vs. 34% (*p* < 0.001) of patient on tablet(s) forgot to take their medication more frequently than those on liquid LT4; in addition, 76.2 vs. 38.1% (*p* < 0.001) of patients on tablets felt hassled about sticking to their treatment plan, and 76.2 vs. 38.1% had difficulty (usually or always) remembering to take all their medication(s), respectively.

**Table 1:** The MMAS-8 questionnaire.

	**Tablet LT4 (161 pz)**	**Liquid LT4 (159 pz)**	***p***
	YES/NO	YES/NO
1. Do you sometimes forget to take your [health concern] medication(s)?	104/57	55/104	<**0.001**
2. People sometimes miss taking their medications for reasons other than forgetting. Thinking over the past 2 weeks, were there any days when you did not take your [health concern] medication(s)?	3/158	4/155	0.722
3. Have you ever cut back or stopped taking your medication(s) without telling your doctor, because you felt worse when you took it?	5/156	2/157	0.448
4. When you travel or leave home, do you sometimes forget to bring along your [health concern] medication(s)?	14/147	20/139	0.281
5. Did you take your [health concern] medication(s) yesterday?	160/1	159/0	0.999
6. When you feel like your [health concern] is under control, do you sometimes stop taking your medication(s)?	3/158	4/155	0.498
7. Taking medication(s) every day is a real inconvenience for some people. Do you ever feel hassled about sticking to your [health concern] treatment plan?	116/45	63/96	<**0.001**
8. How often do you have difficulty remembering to take all your medication(s)?			<**0.001**
Never/rarely (4)	0.0%	0.3%
Once in a while (3)	0.0%	0.3%
Sometimes (2)	3.4%	2.8%
Usually (1)	23.4%	7.1%
All the time (0)	38.6%	24.1%

**Figure 1:**
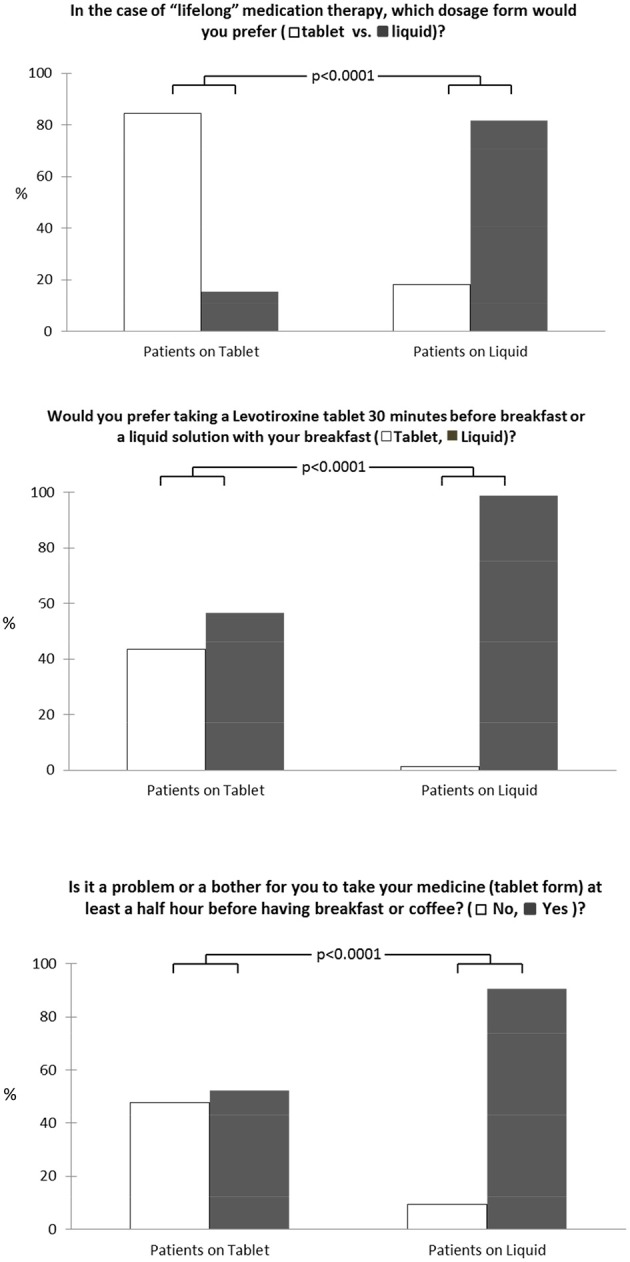
Medication preference for tablet and liquid LT4 formulations for lifetime treatment among our patients.

The 3 items administered to the patients to specifically evaluate medication preference between tablet and liquid LT4 formulations for lifetime treatment are reported in Figure 1. In detail, 165 of 320 patients (51.6%) preferred tablets for lifetime medication therapy even though taking the tablet at least 30 min before breakfast was a problem for 228 of 320 patients (71.2%). For this reason, liquid LT4 ingested with breakfast was the best choice for 248 of 320 patients interviewed (77.8%).

## Discussion

The result of this observational study clearly showed high therapeutic compliance (87% of participants) among patients on tablets and on liquid LT4 formulations, although significant differences emerged. In particular, although half of the patients generally preferred tablets for lifetime therapy, the possibility of taking the same drug with breakfast, but in a liquid form, represents a great improving in daily pharmacologic treatment.

LT4 treatment is one of the most frequent therapies worldwide. It is generally considered straightforward even though many surveys on hypothyroidism show that 40–50% of patients are either over- or undertreated (2, 6). Many factors can explain this “failed” treatment, such as interference with intestinal absorption of LT4 from food, drugs, and disease (15), but also poor medication adherence. This last point is important not only because it represents the main cause of low therapeutic efficacy but also affects health care costs (16). Patient questionnaires are widely used to measure adherence to therapies in the clinical setting due to their simplicity and low cost. The MMAS is one of the most frequently used patient questionnaires for the assessment of medication adherence (17). The latest version consists of 8 items, the first 7 are yes/no questions and the last is a 5-point Likert scale rating (14). Since its development, the MMAS-8 has been used in more than 200 randomized controlled trials of medical adherence interventions regarding numerous chronic diseases (18). The Italian version has recently been validated in a survey investigating the management of newly diagnosed hypothyroidism compared with the recommendations of the American Thyroid Association, American Association of Clinical Endocrinologists, and European Thyroid Association guidelines (19).

Few data are available on therapeutic adherence in patients with hypothyroidism with heterogeneous results. Briesacher et al. (20), using health care claims data, reported therapeutic adherence of 36.8%, whereas Crilly and Esmail (21) reported 78% adherence, similar to our data. This result was also observed by Vezzani et al. (19) who reported medium-high adherence in 76.1% of patients with hypothyroidism. Moreover, these percentages were confirmed among pregnant women with hypothyroidism (22).

In a large retrospective study, it has been shown that different LT4 formulations can have an impact on therapeutic compliance (23). Our study does not confirm these data; no differences between patients taking tablet and liquid LT4 formulations were seen. This was also recently demonstrated in the EDIPO study in which no correlation was seen between adherence to treatment and LT4 formulation (19). Although a large cohort of patients was included in the retrospective survey by Hepp et al. (23), adherence was calculated from the Truven's Health Analytic MarcketScan Commercial Claim database, which consists of the health care records of millions of commercially insured individuals. Thus, therapeutic adherence was calculated only from medical claims linked to drugs prescriptions (which could be changed for many reasons) and not from the “feeling of therapeutic adherence” reported by the patients. In contrast, in our study and in the EDIPO study, we prospectively evaluated the MMAS-8, a validated questionnaire widely used to measure adherence to therapies; we found a high adherence independent of the LT4 formulation. However, patients taking LT4 in tablet form forgot their medication more frequently (*p* < 0.001), felt hassled about sticking to their treatment plan (*p* < 0.001), and had difficulty remembering to take all their medication(s) (*p* < 0.001) than those on liquid LT4.

Guglielmi et al. (24) showed that changing from LT4 tablets to the liquid formulation at breakfast improved the quality of life of the patients “resulting in the perception of a greater convenience of the treatment.” Our study confirms and underlines this important aspect of “real” life. Even though half of the patients generally preferred tablets for lifetime therapy, taking medication at least 30 min before breakfast was a problem for most of them. For this reason, the possibility of taking a liquid formulation at breakfast was the best choice for two thirds of the patients interviewed.

In conclusion, our study demonstrated high adherence to LT4 treatment in both tablet and liquid form. The possibility of taking LT4 with breakfast was the most convenient option for most of the patients.

## Ethics statement

All data collected will remain strictly confidential and anonymous in accordance with ethical rules of our University and Hospital Institutions.

## Author contributions

CC: Concept and design. RC, FM, AP, MM, MS, EG, and AC: Acquisition of data. CC and IP: Analysis and interpretation of data. CC: Manuscript writing. AF and MC: Review of final manuscript.

### Conflict of interest statement

The authors declare that the research was conducted in the absence of any commercial or financial relationships that could be construed as a potential conflict of interest.
